# Implementation of breast cancer continuum of care in low- and middle-income countries during the COVID-19 pandemic

**DOI:** 10.2217/fon-2020-0574

**Published:** 2020-07-27

**Authors:** Hagar Elghazawy, Joaira Bakkach, Mohamed S Zaghloul, Atlal Abusanad, Mariam Mohamed Hussein, Mohamed Alorabi, Nermean Bahie eldin, Thanaa Helal, Tarek M Zaghloul, Bhanu Prasad Venkatesulu, Hesham Elghazaly, Sana Al-Sukhun

**Affiliations:** ^1^Department of Clinical Oncology, Faculty of medicine, Ain Shams University Hospitals, Cairo 11566, Egypt; ^2^Biomedical Genomics & Oncogenetics Research Laboratory. Faculty of Sciences and Techniques of Tangier. Abdel Malek Essaadi University, Tangier 90000, Morocco; ^3^Department of Radiotherapy, National Cancer Institute, Cairo University, Cairo 12622, Egypt; ^4^Department of Medicine, Oncology Division, King Abdulaziz University, Jeddah 23221, Saudi Arabia; ^5^Department of Pathology, Ain Shams University Hospitals, Cairo 11566, Egypt; ^6^Department of Surgical Oncology, National Cancer Institute, Cairo University, Cairo 12622, Egypt; ^7^Department of Internal Medicine, Henry Ford Hospital, Detroit, MI 48202, USA; ^8^Al-Hyatt Oncology Center, Faculty of medicine, Jordan University, Amman 11183, Jordan

**Keywords:** breast cancer, coronavirus, COVID-19, low- and middle-income countries, pandemic, SARS-CoV-2

## Abstract

Breast cancer is the most common malignancy among women worldwide. The current COVID-19 pandemic represents an unprecedented challenge leading to care disruption, which is more severe in low- and middle-income countries (LMIC) due to existing economic obstacles. This review presents the global perspective and preparedness plans for breast cancer continuum of care amid the COVID-19 outbreak and discusses challenges faced by LMIC in implementing these strategies. Prioritization and triage of breast cancer patients in a multidisciplinary team setting are of paramount importance. Deescalation of systemic and radiation therapy can be utilized safely in selected clinical scenarios. The presence of a framework and resource-adapted recommendations exploiting available evidence-based data with judicious personalized use of current resources is essential for breast cancer care in LMIC during the COVID-19 pandemic.

After the formal declaration of the coronavirus disease-19 (COVID-19) as a pandemic by the WHO, on 11 March 2020 [1], the world has been facing unprecedented healthcare challenges in all disciplines. Until 20 April 2020, the total number of infected cases exceeded 2,300,000 worldwide, of which 157,000 died. Comparing these figures with the approximately 266,000 confirmed cases and 11,000 deaths on 20 March 2020 helps us understand the apparent rapid transmission dynamics of COVID-19 worldwide [2].

This crisis has cast a serious shadow on both cancer patients and cancer care providers. The novel virus (SARS-CoV-2) causing COVID-19 has higher morbidity and mortality in high-risk patients [3,4], including those with cancer. Once infected, this vulnerable group has higher rates of hospitalization, admission to intensive care units, and invasive tracheal intubation [5]. This scenario creates a dilemma for physicians because the delay in delivering cancer-directed care may lead to cancer progression; on the other hand, chemotherapeutic agents and radiation therapy suppress the immune system and make the patients more vulnerable to developing COVID-19.

Although COVID-19 does not differentiate between high-income countries (HIC) and low- and middle-income countries (LMIC), its associated ramifications on healthcare may be more severe in LMIC. The World Bank classifies the world's economies according to their national income per person [6]. The LMIC have a low gross domestic product (GDP), low GDP per capita, low capital expenditure with a small percentage of health expenditure from GDP, low hospital bed numbers and low physician number per 1000 population. Governments have to make difficult decisions regarding priorities for their limited budgets in LMIC. Health workforces are correspondingly small and unable to cope with the burden of cancer [7]. Moreover, some LMIC suffer from civil wars, political turbulence, terrorism and social instability [6,7].

On the other hand, while the global response is directed to mitigate the straining effect of the COVID-19 crisis on the healthcare system, cancer care in LMIC is burdened with imperfect access to care, with long traveling distances to reach oncology centers, detrimental cultural habits and increased financial toxicity [7, 8]. Generally, cancer patients in LMIC face many structural, sociocultural, personal and financial factors that can influence their care, especially women's opportunities to seek and receive care in a normal situation. These are much aggravated with the virus pandemic [9].

Breast cancer (BC) represents the most commonly diagnosed cancer in women worldwide, with an estimated >2 million new cases (11.6% of all new cancer cases) and >626,000 deaths (6.6% of all cancer deaths) in 2018; it is the leading cause of cancer death in women [10]. The absolute burden of BC is higher in less-developed regions, with projected estimates to exceed 1 million new cases/year in LMICs alone by 2020 [11]. Moreover, BC in LMIC has worse survival, mainly due to late presentation of the disease and limited resources [12].

In response to this pandemic, BC specialists are trying to modify care to minimize the patients' exposure risk without compromising oncological outcomes in the light of the best available evidence. Several international societies and organizations have provided recommendations and guidance for triaging, prioritizing and managing BC patients. Nevertheless, these recommendations relate mainly to developed countries’ perspectives on cancer care during the COVID-19 pandemic, which might be challenging to apply to LMIC for different factors. These factors include, but are not limited to, the locally applied health system model and expenditure, facility and staffing ratio for oncology programs, access to care, preexisting cancer care disparities, limited awareness, different community values and in some countries, political instability. Together those issues highlight the importance of a framework addressing urgent needs in the face of strained resources, and the value of resource-adapted recommendations [13] for BC care to be implemented in normal situations (without a crisis) and if facing a crisis (like the current pandemic).

In this review we summarize the recommendations for the BC continuum of care during the COVID-19 pandemic from different global perspectives. The challenges for implementation of such strategies in LMIC will be illustrated. Every country, including LMIC, may adapt the plan that is in line with its current pandemic situation, ensuring that there is no ‘one size fits all’ concept. Also, it is of utmost importance to point to the vital role of the clinician's judgment for the management of individual patients, taking into consideration both the institutional workforce and patients’ values and benefit.

## Methodology

Medline (PubMed) and the Cochrane Library databases were searched to 30 May 2020, using the keywords: ‘COVID-19’ AND (‘Breast cancer’ OR ‘Carcinoma of the breast’). Evidence-based guidelines from international societies were also reviewed. The search was limited to English-language literature. A total of 78 articles were identified. All BC care and COVID-19 pandemic publications were reviewed thoroughly to cover all aspects of the BC continuum of care from around the globe, taking into consideration that each country has different resources at different phases of the pandemic. It was meant to help in weighing the consequences of the viral infection against the benefit of receiving the preplanned treatment for BC patients, based on the best available evidence. Also, challenges and future perspectives for providing the best oncological services in LMIC were highlighted.

## Results

### BC patients’ vulnerability to COVID-19

According to the CDC, cancer patients, individuals aged ≥65 years and with chronic diseases (e.g., chronic lung diseases, serious heart conditions, severe obesity, diabetes and liver disease) are at higher risk for severe illness from COVID-19 and require extra precautions [14]. The association between cancer and COVID-19 infection is based on the assumption that these patients are immunocompromised due to the disease or the treatments they receive. Current evidence for this assertion is provided by many studies reporting increased risks and worse outcomes for cancer patients [3,15,16].

The first study to address the clinical outcomes of cancer patients infected with COVID-19 was a retrospective report by Zhang *et al.* [15], which identified 28 infected cancer patients among 1276 COVID-19 patients admitted to three hospitals in Wuhan. The prevalence (2.2%) was 1.7-times higher than in the Chinese population of the same age [17]. Despite the small sample size of cancer patients, the report provided three essential points: first, the mortality rate estimate (28.6%) for the infected cancer patients was at least ten-times higher than that reported in all COVID-19 patients in China; second, the recent use of anticancer therapies within 14 days of infection (including chemotherapy and radiation) was an independent predictor of death or other severe events with a hazard ratio >4; and third, a high proportion of patients acquired the infection while already in the hospital for cancer treatment (28.6%). Moreover, 19.5% of those who died from COVID-19 in Italy had active cancer in the last 5 years, as per a report published in March 2020 [18].

There is still a gap in knowledge of the distribution of histologic subtypes in COVID-19 cancer patients, and the specific risk for BC patients is yet to be clarified. Available data suggest that men are more susceptible to COVID-19 [19], but women do still become infected; BC patients with multiple risk factors (e.g., diabetes, hypertension, preexisting cardiovascular diseases) are especially vulnerable to the viral infection [14]. The proportion of patients with BC ranged from 8.3% to 29% in published studies [15,20]. In an Italian series of cancer patients infected with COVID-19 in a 1-month duration, BC patients represented five of 17 patients, of whom two were on chemotherapy, one was on adjuvant endocrine therapy and two patients were on mTOR inhibitors or an anti-Her2 agent in the metastatic setting [20].

Interestingly, the most prevalent malignancy was BC (21%) in the international COVID-19 and Cancer Consortium registry that collected data on 928 patients with active or previous malignancy who had confirmed COVID-19 infection over a 1-month period [21]. Furthermore, worse outcomes from COVID-19 infection are increasingly being reported in patients with cancer, particularly those with older age, active cancer, metastatic disease or other comorbidities [22]. These data altogether support prompt actions toward protecting BC patients who may have more than one risk factor for COVID-19 infection or complications.

### The global recommendations for BC continuum of care during COVID-19

As the overall pandemic situation continues to evolve, BC oncologists and surgeons are forced toward deferring or deescalation strategies. International communities (e.g., NICE, NCCN, ESMO, ACS, SSO) [23-31] stress the importance of continuing oncology care, especially in the curative setting, while deferring other measures until the pandemic curve is flattened. Deferring cancer care as an alternative until the end of the pandemic may seriously jeopardize survival and compromise clinical outcomes in the future [30]. The possible medicolegal consequences of delaying treatment without definite widely adopted and documented guidelines add to the challenge.

### General management of the crisis & infection control measures

Adopting the appropriate crisis management plan is of paramount importance. Such a plan should include five main components: leadership and communication, patient management, staff management, infection control and recovery plan. Dealing promptly with these components can result in the prevention of any new infection, with zero in-hospital transmission of the viral infection among oncology patients, not just during the months of the crisis, but over the subsequent years [32,33]. Highlights of the mandatory measures are illustrated below.

#### Communication and infection control

- Clear communication, with written documentation for multidisciplinary team (MDT) discussions (the rationale behind every treatment) and discussion of the risks and benefits of therapy with patients and their relatives, is fundamental [23,24];
- Adequate disinfectants must be available at the hospital entrance and the radiotherapy facility [30].

#### Patient management

- Advise cancer patients to use face masks outside their homes, especially in health facilities and if having chest symptoms (cough, sneezing), and employ frequent hand washing protocols [30,31,34,35];
- Arrange waiting areas to keep a distance of 1–2 m between patients[119];
- Use telemedicine and online consultation services if possible [36];
- Consider calling outpatients by telephone 1 day before their appointments to screen for COVID-19 symptoms and history of contacting COVID-19-positive patients [37];
- Triage and screen. All patients scheduled for hospital visits should be screened for signs of COVID-19; suspected patients should be isolated, evaluated thoroughly and referred to specialized COVID-19 caring centers [37];
- ‘No visitors’ policy unless there is a particular need [37];
- Consider the COVID-19 screening test for patients who need hospitalization [38] and before every chemotherapy cycle [39];
- Consider possible causes other than COVID-19 in patients with new onset of respiratory symptoms [40], such as influenza, bacterial pneumonia, treatment-related side effects (e.g., atelectasis, pulmonary embolism, pneumonitis) or tumor progression (e.g., lymphangitis carcinomatosis);
- Cautiously assess new lung infiltrates on radiographic imaging. It may be difficult to distinguish pneumonitis secondary to antineoplastic agents (e.g., mTOR inhibitors or checkpoint inhibitors) from disease progression or viral infection. In this case, therapy should be withheld until COVID-19 infection is excluded [40];
- Once the patient is suspected to have COVID-19 based on symptoms or history of contact with a known positive individual, a cancer treatment break is recommended till cleared by a negative test result [39];
- It is still unknown when cancer treatment can be safely resumed after COVID-19 infection, given the unknown effects of further manipulation of the patient's immune system quickly after the viral infection and the undocumented reinfection rates. Withholding cancer treatment until COVID-19 symptoms have resolved for at least 72 h with a negative viral load test, and considering a second consecutive negative test within 48 h, is the accepted approach by many authors [23,40,41];
- Limit hospital visits for elderly cancer patients unless urgently needed. Higher case fatality rates with increasing age (overall case fatality rate of 2%, rising to 15% for age ≥80 years) and with comorbidities (11% for cardiovascular disease, 7% for diabetes, 6% for chronic respiratory disease) have been reported [42].

#### Staff management

- Healthcare workers are at high risk of being infected with the virus; at least 2629 were infected, of whom 8.3% died, since the onset of the outbreak in Italy [43];
- Use extensive infection control measures and personal protective equipment when dealing with infected patients [44,45];
- Reduce direct communication by using cyberspace capacities to communicate with each other and the patients, and perform virtual MDT meetings [37];
- Divide BC teams (surgeons, medical and radiation oncologists, technicians) into tandem operation teams (ideally 50% on-site, 50% off-site) to reduce risk of exposure to infection with ideally regular alternation every 2 weeks (to overcome the incubation period of the 14-day half-life of SARS-CoV-2) [30];
- Supply pathologists who perform fine-needle aspiration of breast lesions with enhanced biosafety precautions (i.e., procedure room with adequate ventilation, hand hygiene, wearing personal protective equipment including surgical mask, eye protection and gown) if possible, because they may be at increased risk for exposure to infected droplets [46];
- Extend the infusion unit and radiation therapy working hours from early morning to late evening, to accommodate any delayed patients with appropriate distancing and isolation [32];
- Support the presence of organizations that can provide oncological home care, applying ‘double triage’ protocol to ensure the continuity of care and protect healthcare professionals from the exposure to infection [47];
- Allocate older healthcare workers and those with comorbidities to off-site duties, if possible [42].

### BC patients triage & prioritization

Triaging BC patients by MDT is of utmost importance, keeping workload balance between different disciplines [24]. BC patients should be prioritized into three priority levels (1, 2 and 3) according to the urgency of their cancer care [24-27,48-50]:

- Priority 1: patients with immediately life-threatening conditions or symptoms requiring urgent treatment, even if limited resources, with the aim of preservation of life or symptomatic relief;
- Priority 2: patients who do not require immediate treatment but should start during the pandemic, providing that a short delay (≤3 months) would not impact the overall outcome. Most BC patients are in this priority group but further stratification is needed;
- Priority 3: patients whose treatment can be safely deferred until the pandemic peak ends (delay for more than 3 months) with no substantial impact on the long-term outcome.

Factors that must be taken into consideration when adapting these priority levels are the individual risk of COVID-19 infection according to age and comorbidities, the hospital capacity, the severity of the COVID-19 pandemic in each country, the stage of the malignancy along with its biology and risk of progression affecting the survival [24,28,38].

### Prioritization of different steps in BC management

#### Screening, imaging & workup

##### Priority 1

- Breast imaging for urgent situations such as acute breast abscess or evaluation of severe postoperative complications [24,50];
- BC diagnosis during pregnancy [25].

##### Priority 2

- Breast diagnostic imaging for suspicious breast symptoms, biopsies for BIRADS 4–5 (Breast Imaging-Reporting and Data System) lesions and breast MRI for the extent of disease evaluation or prechemotherapy assessment [24,25,50];
- Evaluation of symptomatic metastatic relapse, while metastatic BC patients can be followed clinically (symptom-oriented and by examination) [24,25].

##### Priority 3

- Postponing BC screening for the asymptomatic population. It can be rescheduled after the pandemic with no probable adverse impact on survival [24,25,37,41,50,51]. However, symptomatic patients need to be referred for evaluation;
- Evaluation of BIRADS 3 lesions can be postponed [24,25,50];
- Metastatic workup in early-stage asymptomatic BC patients [24,25];
- Bone density evaluation when using aromatase inhibitors [24,25].

#### Surgery

The uncertainty regarding when the pandemic will end is one of the main challenges of delaying the definitive surgery [38].

##### Priority 1

- BC surgery complications (e.g., bleeding, breast abscess) or reconstruction complications, such as ischemia [24-28,50];
- Patients who completed neoadjuvant chemotherapy-based treatment with accepted delay of surgery (≤8 weeks post-chemotherapy) have no impairment of BC outcomes [24,27,28];
- Upfront surgery is preferred in the following conditions:
  - cT1 N0, triple negative BC (TNBC) and HER2-overexpressing (HER2+) patients [26,27];
  - Patients who showed tumor progression during neoadjuvant treatment should be referred for surgery if the tumor is resectable [24,26-28];
  - Resectable BC categories that receive neoadjuvant therapy according to standard approach (TNBC, HER2+ tumors and ≥cT2 or N+ Estrogen Receptors positive (ER+)/HER2− tumors) can be directed for upfront surgery if the pandemic is not in an escalatory phase with currently available hospital resources that are likely to be occupied in the next weeks/months [26,28,50];
- Immediate reconstruction with implant or tissue expanders can be performed only if hospital resources permit or if toilet mastectomy is required in a fungating tumors to close the skin [24,50];
- BC surgery during pregnancy (multidisciplinary treatment should be individualized according to stage and biology) [25,28];
- Breast angiosarcoma and malignant phyllodes tumors [27].

##### Priority 2

- Low-risk BCs (e.g., stage I-II, ER+/PR+, HER2−, low-grade, low-proliferative index) have lower priority because they can be considered for starting preoperative endocrine therapy and for deferring surgery until it becomes possible [24,25,28].

##### Priority 3

- Excision of benign lesions and duct excision (fibroadenomas, atypia, papillomas) [24-27,50];
- Surgery for ER+ ductal carcinoma *in situ* (DCIS), considering ER− DCIS as a higher priority for surgery (priority 2) [24,25,27,28];
- Autologous breast reconstruction surgeries should be deferred [24-27];
- Reexcision surgery [24-27];
- Prophylactic surgery for asymptomatic high-risk patients [24-27,50].

#### Medical oncology

Tailored recommendations for systemic therapy in BC patients during the COVID-19 pandemic according to treatment setting and BC biology are shown in Table 1A & 1B.

**Table 1A.** Tailored recommendations for systemic therapy in breast cancer patients during the COVID-19 pandemic according to treatment setting.

Treatment setting	Recommendations	Notes	Ref.
Neo/adjuvant setting	Chemotherapy should be administered for 12–24 weeks (four–eight cycles) Sequential anthracycline (ANT)/taxane-based regimen is the standard for the majority of patients In selected lower-risk patients, four cycles of ANT- or taxane-based chemotherapy or CMF may be used Consider modification of chemotherapy schedules to reduce clinical visits (3 weeks dosing instead of weekly when appropriate) ANT-based regimens preferably not including 5-FU (EC or AC are the standard)	Phase III trial showed that 5-FU fails to improve the efficacy of ANT-based regimens and increases toxicity	[24,25,28,50]
Metastatic setting	Consider modification of chemotherapy schedules to reduce clinical visits (3 weeks dosing instead of weekly when appropriate) Consider shifting to oral agents when appropriate Consider treatment holiday for controlled disease		[24,25,28,50]

AC: Adriamycin and cyclophosphamide; ANT: Anthracyclines; BC: Breast cancer; CMF: Cyclophosphamide, methotrexate, fluorouracil; DCIS: Ductal carcinoma *in situ*; DM1: Ado-trastuzumab emtansine; EC: Epirubicin, cyclophosphamide; ER: Estrogen receptor; LCIS: Lobular carcinoma *in situ*; LHRH: Luteinizing-hormone releasing hormone; PD-L1: Programmed death-ligand 1; TNBC: Triple-negative breast cancer; 5-FU: 5-fluorouracil.

**Table 1B.** Tailored recommendations for systemic therapy in breast cancer patients during the COVID-19 pandemic according to tumor biology.

Breast cancer biology	Recommendations	Notes	Ref.
Atypical hyperplasia− ER+ LCIS− ER+ DCIS	Effectively treated with either tamoxifen or aromatase inhibitors ER+ DCIS: consider preoperative endocrine therapy for 6 months		[24,25,27,28]
Low-risk luminal BC (stage I–II, ER+/HER2−, low-grade, lobular BC, oncotype score <25, luminal A signature)	Consider neoadjuvant endocrinal treatment for 6–12 months or till maximum response Consider omitting adjuvant chemotherapy	These low-risk patients do not usually benefit from chemotherapy	[24,25,26,50]
Premenopausal BC patients who are planned for LHRH agonists	Consider applying /3 months dosing, to reduce patient visits Consider monthly home administration by the patient or visiting nurse		[24,50]
Non-metastatic HER2+ BC	Should be treated with chemotherapy and anti-HER2 therapy T1aN0: consider omission of chemotherapy and anti-HER2 therapy Low-risk or elderly patients with cardiovascular or other comorbidities: consider discontinuation of anti-HER2 agents after 6 months instead of 12 months of treatment Stage I: consider substituting trastuzumab-DM1 instead of paclitaxel/trastuzumab Consider delaying cardiac monitoring (echo etc.) during anti-HER2 therapy, for clinically stable patients	Anti-HER2 agents (trastuzumab and pertuzumab) should be safe to use; they are unlikely to affect immune function Based on data from prospective randomized trials Based on randomized trial favoring patient safety	[24,25,50]
TNBC	Platinum should not be used routinely in the adjuvant setting Consider chemotherapy plus atezolizumab in PD-L1+ advanced TNBC after weighing benefits vs risks	Close monitoring for specific symptoms of infection or pneumonitis	[25,28]
Metastatic HER2+ BC patients	Consider early introduction of anti-HER2 agents (e.g., pertuzumab/trastuzumab) Consider giving anti-HER2 agents at longer intervals (i.e., /4 weeks) Consider discontinuation of maintenance anti-HER2 agents (e.g., trastuzumab, pertuzumab) for patients with minimal burden and disease control for ≥2 years	This is more likely to improve outcomes without impact on the immune system.	[24,25,50]
Metastatic ER+HER2− BC patients	Consider delaying adding CDK4/6, mTOR, or PIK3CA inhibitors to endocrine therapy, especially in elderly patients with comorbidities, in the following situations: 1. Where endocrine-therapy alone is providing effective tumor control 2. In first line 3. Low disease burden or bone-only disease Dose reduction of palbociclib does not decrease efficacy	Use of these agents must be weighed against the increased risk of adverse events (e.g., neutropenia, pneumonitis) Endocrine therapies can be safely used (e.g., tamoxifen, aromatase inhibitors, fulvestrant) with no effect on immune function Fulvestrant needs monthly application	[24,25,50]

AC: Adriamycin and cyclophosphamide; ANT: Anthracyclines; BC: Breast cancer; CMF: Cyclophosphamide, methotrexate, fluorouracil; DCIS: Ductal carcinoma *in situ*; DM1: Ado-trastuzumab emtansine; EC: Epirubicin, cyclophosphamide; ER: Estrogen receptor; LCIS: Lobular carcinoma *in situ*; LHRH: Luteinizing-hormone releasing hormone; PD-L1: Programmed death-ligand 1; TNBC: Triple-negative breast cancer; 5-FU: 5-fluorouracil.

##### Priority 1

- Patients with oncological emergencies requiring immediate treatment (e.g., febrile neutropenia, intractable pain, pleural effusion) [24];
- Neoadjuvant therapy is already considered as the standard of care for non-metastatic ≥ cT2 N0 TNBC, HER2+ BC and inflammatory BC based on the higher rates of clinical and pathological tumor response showing durable tumor control before surgery [24,25,28,50,52];
- Standard adjuvant systemic therapy for TNBC and Her2+ BC [24,25,28,50];
- Continuation of adjuvant capecitabine treatment in TNBC patients and ado-trastuzumab emtansine (T-DM1) in HER2+ BC patients (in case of residual disease in post-neoadjuvant setting) [25,28];
- Completion of standard adjuvant endocrine therapy [24,25,28,50].

##### Priority 2

- Early introduction of systemic therapy for metastatic BC, more likely to improve outcomes [25,28];
- Patients with ER+, HER2− tumors can defer surgery and receive neoadjuvant endocrine therapy for 6–12 months without clinical compromise [24,25,28].

##### Priority 3

- Later metastatic lines of therapy less likely to improve outcomes [25,50];
- Anti-resorptive bone-targeted therapy (zoledronic acid and denosumab) could be withheld in the adjuvant and metastatic setting or used with a 3-month interval, except when strictly needed for hypercalcemia [24,25,28,50].

#### Radiation therapy

Evidence suggests that COVID-19 is spread by droplets with an incubation period of 1–14 days [48]; some researchers have raised concerns that SARS-CoV-2 particles might remain viable for up to 72 h, which has implications for most radiation activity and equipment [53].

As long as sufficient protective gear is available, surgical masks should be worn [54] and staff should quickly disinfect after every patient (handwashing, breast boards), without interfering with the planned schedule. When possible, hypofractionated regimens should be strongly considered [24,28,48,^50^] and care should be taken that BC patients receiving adjuvant radiotherapy (RT) who present with chest symptoms (cough, dyspnea) should be promptly evaluated. Tailored recommendations for RT techniques in BC patients during the COVID-19 pandemic are shown in Table 2.

**Table 2.** Tailored recommendations for radiation therapy techniques in breast cancer patients during the COVID-19 pandemic.

Breast cancer population/setting	Recommendations	Notes	Ref.
Partial breast irradiation (EBRT)	For appropriate patients (ASTRO criteria) IMRT: consider 3DCRT Follow dose constraints: 6 Gy/5 Fr, daily 5.7 Gy/5 Fr, daily 3.8 Gy/10 Fr, BID	High-level evidence supports equal or superior survival versus WBRT Equivalent local control versus WBRT	[55,56,57,58,59,60]
Partial breast irradiation (IORT)	For appropriate patients (ASTRO criteria) 20 Gy once		[56,61]
WBRT +/- regional lymph nodes	• Hypofractionation: 2.67 Gy/15 Fr, daily, 3DCRT • Extreme hypo-fractionation: For appropriate patients (node negative, boost not needed) Consider 5.7 Gy/5 Fr, weekly Consider 5.2 Gy/5 Fr, daily • Regional nodal irradiation: Consider omission in low risk (Z011 criteria), allowing for less complex techniques	High level evidence supports long-term efficacy and safety While data suggest early toxicity and equivalent 3 years local control, the long-term local control results are pending	[57,62,63,64,65,66]
Chest wall +/- regional lymph nodes	2.67 Gy/15 Fr, daily, 3DCRT 2.9 Gy/15 Fr, daily, 3DCRT 2.5 Gy/15 Fr, daily, 3DCRT	No difference in efficacy or toxicity versus conventional RT	[67,68]
Deep inspiratory breath hold techniques	Avoid active breathing control due to risk of aerosol contamination Voluntary breath-holding techniques can be used		[69]
Brain metastasis	SRS (15 Gy/1 Fr) for 1–3 metastases, good KPS, no extracranial disease. 3D whole-brain RT (4 Gy/5 Fr)	No confirmed survival benefit versus observation	[48] [70,71]
Bone metastasis	With or without cord compression: 8 Gy/1 Fr Pathological fracture: 4 Gy/5 Fr	No survival difference between different fractionations; controversy in pain flare	[72,74] [75]

BID: Twice a day; EBRT: External beam radiotherapy; Fr: Fraction; IMRT: Intensity modulated radiotherapy; IORT: Intraoperative radiotherapy; KPS: Karnofsky Performance Status; RT: Radiotherapy; SRS: Stereotactic radiosurgery; WBRT: Whole breast radiotherapy; 3DCRT: 3D conformal radiotherapy.

##### Priority 1

- Palliative treatment of bleeding/fungating inoperable breast mass, spinal cord compression and symptomatic brain metastases if controlling the symptoms cannot be achieved by other measures [28,50,76];
- Patients already on adjuvant RT [28,50];
- Adjuvant postoperative RT within 2–4 months post surgery, for high-risk BC patients (inflammatory BC, lymph node positive, TNBC or HER2+, residual disease after neoadjuvant therapy, young age [<40 years]) [28,50,77].

##### Priority 2

- Adjuvant postoperative RT within 5–6 months post surgery for low/intermediate-risk BC patients (age <65 years and stage I–III luminal cancer, or positive margins), with starting endocrinal therapy [28,50,78].

##### Priority 3

- After breast-conserving surgery:
  - Low-risk elderly (≥65 years): omit or defer whole-breast radiation (WBRT) for stage I, ER+/HER2− receiving adjuvant endocrine therapy, without impacting survival (risk of COVID-19 infection outweighs benefit of RT) [24,50,62,79,80];
  - DCIS: omit WBRT, especially for those with ER+ disease receiving adjuvant endocrine therapy, without affecting overall survival [24,50,81];
  - Invasive disease with low-risk genomic profile: consider omission of WBRT cautiously; trials are ongoing (e.g., LUMINA, IDEA, PRECISION) [48];
  - Boost: omit the boost in invasive disease (except for patient ≤40 years of age or with positive margin) [82-84] and *in situ* (except for positive margin and where reexcision is not possible) [24]; there is no survival benefit except for high-risk disease. If given, the boost should be planned simultaneously or as integrated [62].
- After mastectomy:
  - T1-2 N+: consider omission of RT cautiously; trials are ongoing (NSABP B-51, RTOG 1304) [48].
- Palliation:
  - Postoperative for pathological fracture: omit RT as there is limited evidence supporting benefit [85].

#### Supportive care

- Currently, there is no evidence to support prophylactic antiviral therapy use for COVID-19 in either the general population or immunosuppressed patients [40,86];
- Given the controversial immune-modulatory effect of corticosteroids, their use should be limited, as appropriate. However, some researchers have reported that corticosteroids might be beneficial if utilized in the early acute phase of infection [25,87];
- Despite some reports raising concerns about the influence of NSAIDs [88] and angiotensin-converting enzyme inhibitors [89] on the risk and severity of COVID-19, there is no high-level evidence to support avoiding them. A single study which investigated NSAIDs showed that indomethacin had antiviral activity [90]. Patients already using angiotensin-converting enzyme inhibitors should continue to receive them, as recommended by many societies [91-93];
- Prophylaxis against neutropenic fever should be considered at a lower threshold of the expected risk (e.g., >10%), with closer monitoring of neutrophil count for at-risk patients [41];
- Consider using granulocyte colony-stimulating factor (G-CSF) cautiously. There are limited data implicating G-CSF in accelerating the cytokine storm in COVID-19 patients. This was shown in a case series of three cancer patients on active systemic therapy and infected with SARS-CoV-2 (including one BC patient on chemotherapy). All patients developed rising neutrophil/lymphocyte ratio and suffered respiratory decline at 72 h after G-CSF administration [94];
- Explain to the patients the common side effects of G-CSF (flu-like symptoms: generalized bone pain, mild fever), to reduce the risks of confusion with COVID-19 symptoms and added stress on the patients and caregivers [37];
- Patients on chemotherapy presenting with fever (+/- respiratory symptoms) should be managed immediately as having neutropenic fever, because it is life-threatening, in parallel with suspecting COVID-19 as appropriate [23];
- Strongly consider empiric oral antibiotics for patients with neutropenic fever who are clinically stable [41];
- Consider extra support for the patients’ mental health, due to the added stress, anxiety and social isolation measures resulting from the COVID-19 pandemic that may overwhelm cancer patients already loaded by psychological stress from cancer management [40].

#### Follow-up & surveillance

- Postpone any clinic visit that can be deferred without risk for the patient’s clinical outcome, including surveillance after completing the adjuvant chemotherapy, endocrine therapy, or follow-up of asymptomatic metastatic patients [40,41].

#### Clinical trials & research

Continuation of treatment in the context of a clinical trial should be individualized provided that the benefits to the patient outweigh the risks, with a possible adaptation of procedures if this can be done without affecting patient safety and study conduct [25]. Unfortunately, the COVID-19 pandemic consequences have not only changed patients’ treatment plans and the pace of clinical trials but also affected the whole oncology community, limiting traveling, causing conference cancellations and hindering opportunities for research and education collaboration [38,95]. Thus every effort to enhance online meetings, teleconferences and updating the BC team regarding COVID-19 or BC news should be encouraged.

### Advance directives for patients with BC & COVID-19 infection

Several published data have suggested that patients with comorbidities, including cancer, are at higher risk of complications and death from COVID-19 [3,15-18]. As a result and in an unfortunate attempt to prevent overwhelming healthcare systems, cancer patients may be assigned ‘do not resuscitate’ orders by default [96]. Such an approach is not justified even in the context of limited resources. Each cancer patient has an individual risk determined by the malignancy stage, biology, prognosis and the patient's values, and all these factors have to be integrated to make the appropriate decision. Therefore an updated advance directives discussion that balances the benefits and risks of invasive and intensive care in case of infection is essential. Preferably, it should be conducted by the treating oncology team to avoid under or over treatment [97]. On the other hand, family members in LMIC often have a more active role in the patient’s management plan and treatment decisions than the patient herself, which complicates the informed consent process and the concept of autonomy [98].

## Discussion

An interplay of several factors will determine the speed of spread of the COVID-19 pandemic, its doubling time and long-term duration. These factors include the mean duration of infectivity of a person, the isolation measures and lockdown status of affected countries, and emerging immunity [99,100]. At this moment, it cannot be predicted when the COVID-19 outbreak will end. However, based on evidence from previous pandemics, complete eradication of a pathogen after its emergence is rarely achieved and further waves of the disease are seen [99]. The process of approving a vaccine or effective antiviral therapy will take time (at least 12–18 months), so it is unlikely to be available soon [100]. Modeling studies predict that the COVID-19 pandemic may take months to peak, indicating that healthcare systems may remain under strain for many months [101]. Therefore planning for the care of patients with cancer in the era of COVID-19 should be a priority.

The impact of the outbreak on oncology services varies across different countries, depending on the local situation of health systems and their adopted measures to withstand the pandemic. In Italy, despite its status as a HIC, all healthcare systems and cancer patients were faced with drastic changes in the organizational processes once the government extended the ‘red zone’ to the whole nation [31]. Likewise, African oncology professionals (where African countries are LMIC, except one [8]) face the challenge of protecting patients and the workforce against infection, with more critical decisions needed to decrease the negative impact of the crisis on patients' outcomes [102]. Their challenge is exacerbated by having more locally advanced diseases, limited resources to support COVID-19 infection control and logistic issues [103].

Another critical point is that symptoms of COVID-19 infection (fever, cough, fatigue, shortness of breath, muscle pain, headache, chest pain and diarrhea) largely overlap with symptoms seen after chemotherapy or targeted therapy. Thus the COVID-19 pandemic has added extra challenges to oncologists’ daily clinical decisions [19] because they need more workforce and time to evaluate individual patients’ symptoms adequately.

During this crisis, BC care delivery poses a major challenge for patients from the perspective of weighing the competing risks of death from BC versus their vulnerability for COVID-19 infection as immuno-compromised patients. Evidence shows that postponing treatment adversely affects cancer care outcomes; in one study, a 4-week delay in the adjuvant chemotherapy for BC was associated with inferior survival, especially for TNBC [104]. Another study including 11,175 BC patients evaluated the impact of delayed treatment on survival, with a median follow-up of 7.9 years. Delayed first treatment (>90 vs ≤30 days post diagnosis) was associated with worse overall survival in patients with non-metastatic BC (hazard ratio: 2.25) and metastatic BC (hazard ratio: 2). Moreover, delayed adjuvant treatment (>90 vs 31–60 days post surgery) was associated with worse survival in patients with non-metastatic BC (hazard ratio: 1.50) [105]. Delay in treatment can be acceptable in HIC, albeit reluctantly, but is not accepted in LMIC given the risk of progression in BC cases that are already in the advanced stages at presentation (60–80% of cases in LMIC) [106], coupled with the limited treatment capacities in LMIC [107]. The advanced stages of BC demand more resources for management than earlier stages, including radiotherapy equipment, more lines of systemic therapy, more clinic visits, palliative care services and psychological support. Compounding the problem in LMIC is the younger age of BC presentation than in HIC, with resultant poorer prognosis, which has ramifications on social and fertility aspects [106].

All the above factors – including the uncertainty of the pandemic’s duration, the strain on resources during the pandemic and the impact of BC treatment delay on survival, along with the known BC situation in LMIC – mandate the implementation of resource-stratified guidelines as previously suggested by Breast Global Health Initiative [7]. Core, limited and enhanced levels of service should be appropriately contextualized or adapted [108], to match the resources of every country. LMIC can mitigate the drastic effects of the pandemic on the survival and oncological outcomes of BC patients by using this stepwise approach. On the other hand, scarce resources during a pandemic should be allocated respecting the four fundamental ethical values proposed by Emanuel *et al.* [109]. These values are: maximizing benefits, treating people equally, providing instrumental value (priority to those who save others) and giving priority to the worst off (giving priority either to the sickest or to younger patients who will have lived the shortest lives if they die untreated). Clinicians should be mindful of misusing age as an arbitrary criterion, disfavoring elderly patients with COVID-19 in resource allocation decisions where there is healthcare shortage. According to the American Geriatrics Society, factors that are predictive of mortality include frailty and severe comorbidities (likely to result in death over a short period of time, such as <6 months), rather than chronological age alone [110].

Not all the recommended measures to face the COVID-19 pandemic globally place extra demands on LMIC. Wearing cloth masks in public and saving surgical masks to be used within the health facilities [39,40] is considered convenient to LMIC. Also, educational materials and programs directed to cancer patients, promoting proper hygiene and infection prevention measures, are feasible.

The financial toxicity of cancer treatments is a recognized issue globally. However, LMIC can overcome the unaffordability of some costly cancer therapies with limited access by enhancing the availability of biosimilars [111]. Trastuzumab plays a pivotal role in BC treatment in both early and metastatic stages. In 2019, WHO included the biosimilars of trastuzumab in its essential medicines list, integrating the previous inclusion of the biosimilars of erythropoietin agents and filgrastim [112]. Currently, there are six approved trastuzumab biosimilars, which have shown equivalent efficacy to trastuzumab [113]. The wide distribution and utilization of such biosimilars in LMIC should be encouraged and not misperceived as merely cheaper options for poorer countries [111].

The distribution of radiotherapy facilities in Africa and Latin America is poor. In Africa, one unit treats approximately 2.47 million patients in LMIC, compared to 0.8 million patients in upper middle-income countries and about 29 countries in these regions lack radiotherapy units altogether [8,114]. This mandates governmental and policymakers’ concerted efforts in planning for the long term.

## Future perspectives

Uncertainty surrounds the future of the COVID-19 pandemic. Several speculations have emerged, including the possibility of manifesting second and third waves, complete eradication with vaccination, diminished viral virulence and the development of herd immunity [99,100,115]. Evidence is still evolving around these theories without a solid conclusion, and several vaccine trials are ongoing [100]. The future course of the pandemic will be revealed as time passes. Until then, important lessons must be learnt, to prepare for any next pandemic or events that may lead to cancer care disruption.

Quality cancer care is often inaccessible, not only in LMIC but also in rural or remote areas of HIC [116]. Measures that support remote access to cancer care, such as virtual clinics and online ordering of medication refills with off-campus means of delivery, have to be integrated into the care delivery model. The use of teleoncology has to be expanded in LMIC, and other applications of medical telecommunications – including pathology, radiology and virtual MDT – have to be utilized to enhance the quality of clinical cancer care [117]. Improving local internet services to cope with the increased utilization of telehealth is also important. Likewise, oncology education and knowledge dissemination can be continued virtually. Recognized annual oncology conferences like ASCO, ESMO breast, and AACR have been conducted virtually, with massive online attendance. Such an occasion gave a more accessible and feasible way to attend major conferences without increasing expenses and travel burdens. This has to continue to boost the participation of oncology professionals from LMIC, even after the pandemic resolves.

Many cancer patients have faced delays in diagnosis and treatment, while some have received alternative treatments instead of the guideline standards. The outcomes for those with such interruption and modification have to be addressed scientifically. Closer monitoring and earlier intervention may balance the consequences of the deviation from the standards of care during the pandemic. Funding and resource allocation for scientific research to address this issue is recommended in the future. We encourage international and local cancer societies to endorse specific guidelines for cancer treatment in times of crisis or disaster such as the COVID-19 pandemic, to be updated periodically, with the training of medical staff on such situations to be ready for the future.

Although strained resources are a common challenge in LMIC and are further stressed by the pandemic situation, there will be a better understanding of the prioritization of cancer treatment and better allocation of the available healthcare resources, especially in LMIC. Future strategic plans to reduce redundancy and bureaucracy in healthcare systems are highly needed. The need to develop resilience and flexibility to adapt alternative strategies is a lesson that should have been learnt from the pandemic situation, motivating the development of future plans to face the upcoming challenges, whether related to this pandemic or different ones.

Moreover, the current pandemic has further highlighted the urgent need to create an advance directive with establishing triage committees. Advance care and planning discussions should occur before patients are in crisis, with the aim of identifying people who do not wish to receive intensive care, including mechanical ventilation [110].

Finally, detailed BC registries are of ultimate importance to assess every country's disease burden, which will guide disease prioritization and allocation of resources for BC care [118], along with longitudinal registries for BC patients to capture COVID-19 infection behavior.

The present review aims to assist BC oncology professionals and stakeholders in LMIC by using compiled data to generate a framework matching the local resources and needs. Applying MDTs to prioritize BC patients efficiently and discuss individual patients’ management plans is recommended. Also, individual LMIC should provide specific resource-stratified guidelines and recommendations for the ethical dilemmas that can be faced while treating BC patients infected with COVID-19, based on local cultural, legal and religious backgrounds.

## Conclusion

We highlight the importance of directing focused efforts and financial support for implementing resource-stratified guidelines to plan BC care in LMIC during the COVID-19 pandemic. Deliberate attention should be given to BC patients, especially those with comorbidities or metastatic disease and those receiving active treatment.

**Executive summary**

**Introduction**

- The world has been facing unprecedented healthcare challenges caused by COVID-19, which are more aggravated in low-and middle-income countries (LMIC).
- Breast cancer (BC) in LMIC is characterized by late presentation, worse survival and younger age groups, along with limited resources obscuring the delivery of quality cancer care.
- During this crisis, BC care delivery poses a major challenge for patients from the perspective of weighing the competing risks of death from BC versus their vulnerability for COVID-19 infection as immuno-compromised patients.

**BC patients’ vulnerability to COVID-19**

- Accumulating evidence shows that cancer patients, particularly those with older age, active cancer, metastatic disease and those with other comorbidities, are at higher risk of infection and worse outcome of COVID-19.

**General management of the crisis & infection control measures**

- Adopting the appropriate crisis management plan can result in the prevention of any new infection, with zero in-hospital transmission of the viral infection among oncology patients, not just during the months of the crisis.

**Triage & prioritization**

- Triaging BC patients by MDT is of utmost importance, keeping workload balanced between different disciplines.
- BC patients should be prioritized into three priority levels (1, 2 and 3) according to the urgency of BC care.

**Prioritization of different steps in BC management**

- Deescalation of systemic and radiation therapy can be utilized safely in selected clinical BC scenarios.

**Advance directives for patients with BC & COVID-19 infection**

- An advance directives discussion that balances benefits versus risk of invasive and intensive care in the case of infection is essential.
- It cannot be predicted when the COVID-19 outbreak will end. However, other waves of the disease are expected, which complicates the clinical decisions required for BC care.

**Discussion**

- The BC continuum of care in LMIC during the pandemic mandates the implementation of resource-stratified guidelines as previously suggested by Breast Global Health Initiative.
- Core, limited and enhanced levels of service should be appropriately contextualized or adapted to match the resources of every country, to mitigate the drastic effects of COVID-19 on the oncological outcomes of BC.
- Integrating telecommunications to allow timely access to cancer care and establishing the required infrastructures are fundamental prerequisites during the pandemic.
- Continuing virtual oncology education and sharing knowledge through online participation in major oncology congresses will boost the participation of oncology professionals from LMIC, even after the pandemic resolves.
- Funding for scientific research to identify the future consequences of the pandemic on cancer care in general, and breast cancer specifically, are highly recommended.
- Lessons learned from the pandemic situation should be used to encourage flexibility, reduce redundancy in cancer care and prepare alternative plans in LMIC, should another similar emergency occur.
